# Simulation of a Petri net-based Model of the Terpenoid Biosynthesis Pathway

**DOI:** 10.1186/1471-2105-11-83

**Published:** 2010-02-09

**Authors:** Aliah Hazmah Hawari, Zeti-Azura Mohamed-Hussein

**Affiliations:** 1 School of Biosciences and Biotechnology, Faculty of Science and Technology, Universiti Kebangsaan Malaysia 43600 UKM Bangi, Selangor, Malaysia; 2 Centre for Bioinformatics Research, Institute of Systems Biology (INBIOSIS), Universiti Kebangsaan Malaysia, 43600 UKM Bangi, Selangor, Malaysia

## Abstract

**Background:**

The development and simulation of dynamic models of terpenoid biosynthesis has yielded a systems perspective that provides new insights into how the structure of this biochemical pathway affects compound synthesis. These insights may eventually help identify reactions that could be experimentally manipulated to amplify terpenoid production. In this study, a dynamic model of the terpenoid biosynthesis pathway was constructed based on the Hybrid Functional Petri Net (HFPN) technique. This technique is a fusion of three other extended Petri net techniques, namely Hybrid Petri Net (HPN), Dynamic Petri Net (HDN) and Functional Petri Net (FPN).

**Results:**

The biological data needed to construct the terpenoid metabolic model were gathered from the literature and from biological databases. These data were used as building blocks to create an HFPNe model and to generate parameters that govern the global behaviour of the model. The dynamic model was simulated and validated against known experimental data obtained from extensive literature searches. The model successfully simulated metabolite concentration changes over time (pt) and the observations correlated with known data. Interactions between the intermediates that affect the production of terpenes could be observed through the introduction of inhibitors that established feedback loops within and crosstalk between the pathways.

**Conclusions:**

Although this metabolic model is only preliminary, it will provide a platform for analysing various high-throughput data, and it should lead to a more holistic understanding of terpenoid biosynthesis.

## Background

Biological networks are complex and may consist of hundreds of reactions that directly and indirectly affect each other. In order to understand the relationship between these reactions in detail, it is necessary to view the network as a whole [1]. Computational models of metabolic networks have been developed in order to provide an overview of the biosynthetic processes involved in multiple pathways [2,3]. Using such models, changes in the concentration of each metabolite corresponding to those that occur during normal and perturbed conditions can be simulated and used to determine how such changes may contribute to the entire biosynthetic process. The use of computational models of this type gives researchers an in-depth view of the problems that need to be solved and points to new strategies and alternatives.

Various approaches can be taken to building and simulating a biological pathway model [4]. The most popular way of describing the behaviour of such a model is the Ordinary Differential Equations (ODEs) approach [5-7]. ODEs are typically derived from the Michaelis-Menten equation. They are then embedded into the model through script writing using programs such as SimBiology in Matlab [8] and Gepasi [9] that were developed for building and editing such models. However, choosing the right approach to building a particular biological model relies on the type of biological pathway it is to represent. Typically, there are three categories of biological pathways: gene regulatory networks [10,11], metabolic pathways [6,12-14] and signalling pathways [4,7,15]. The model presented here, which describes the biosynthesis of terpenoids via two independent pathways, one involving mevalonate (MEV) and one involving methylerythritol phosphate (MEP), falls into the metabolic pathways category.

We chose to model the terpenoid biosynthetic network using Hybrid Functional Petri net with extension (HFPNe). This method allows easy modelling of complex biological information using a graphical approach and also allows various types of entities to be modelled together. The technique is derived from the traditional Petri net theory that was first described by Carl Adam Petri in his 1962 PhD dissertation [16]. HFPNe is an extension of the HFPN architecture with extended features resulting from fusion of three extended Petri net techniques, namely Hybrid Petri net (HPN), Dynamic Petri net (HDN) and Functional Petri net (FPN) [17-20]. This technique is suitable for our modelling purposes for several reasons: (a) it is capable of utilising primitive types (Boolean and string); (b) it is able to treat more than one value in an entity; and (c) it is capable of treating object type for complicated bioprocesses. Finally, HFPNe introduces the new notion of a generic entity, as well as processing and enhancing simulation processes for this notion [17].

**Figure 1:**
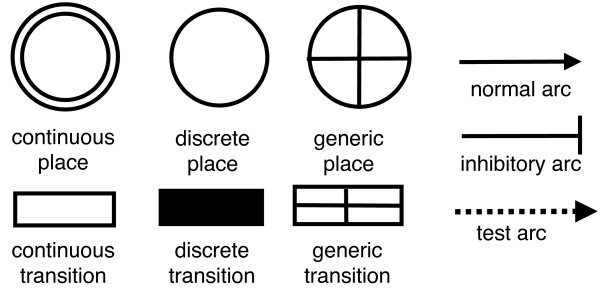
**HFPNe entities**. Places, transitions and arcs are basic entities in the HFPNe architecture. Places and transitions can be subdivided into continuous, discrete and generic.

HFPNe-based biological models are built based on three basic entities called places, transitions and arcs; these are shown in Figure 1[21,22]. In addition to possessing the ability to model continuous and discrete entities (places and transitions) concurrently as described in HDNs, HFPNe introduces generic entities that can hold values in the form of both real number and Boolean strings. This feature enables on/off switches and parameter modulators to be created, which can then function as regulatory components in the model. These HFPNe entities form a network and each is assigned with specific parameters that describe its behaviour, making this technique distinct from other Petri net techniques [10,21-25]. The assignment of specific parameters to the HFPNe entities allows more options to be customised in order to control the behaviour of the model. The parameters of the entities and the topology of their network are generated from biological facts that can be obtained through experimental procedures and by literature and database searches [10,23]. HFPNe and other types of Petri net techniques allow an intuitive approach to modelling; their ability to provide a visual modelling framework for development and simulation [13,14,21,25-29] motivated us to use this technique in our modelling. HFPNe architecture has been successfully applied to the modelling of processes such as vulval development [30] and the operation of microRNA regulatory networks [31] in *Caenorhabditis elegans*.

The biosynthesis of the universal precursors isopentenyl diphosphate (IPP) and dimethylallyl diphosphate (DMAPP) occurs by two independent pathways, the mevalonate (MEV) pathway and the methylerythritol phosphate (MEP) pathway. These two pathways are distinct in terms of organism, cellular localisation and products as well as in the metabolites and precursors involved. The classical pathway, the MEV pathway, was thought to be the only pathway involved in terpenoid biosynthesis until the MEP pathway was discovered in the early 90s [32,33]. The MEV pathway usually operates in the cytoplasm of eukaryotic organisms such as *Gallus gallus *and *Rattus norvegicus*, whereas the MEP pathway takes place in the plastids of eubacteria such as *Escherichia coli *and *Streptomyces cerevisiae*, as well as in the chloroplasts of plants [34-36]. The MEP pathway is not found in animals or fungi, making it attractive for drug design studies because the enzymes in this pathway are using a specific substrate or generate a specific product and their function cannot be compensated for by another enzyme [37,38]. However, both pathways are operational in higher-level plants such as *Arabidopsis thaliana *and *Helianthus annuu*s, as well as in selected eubacteria such as *Streptomyces sp*. [34,39-42].

The several classes of terpenoids (e.g., monoterpenes, sesquiterpenes, triterpenes and diterpenes) are organised according to the number of isoprene units in their backbone. Isoprene units are five-carbon compounds that act as the building block for terpenoids. Isoprenes (C5) consist of one isoprene unit, monoterpenes (C10) consist of two isoprene units, sesquiterpenes (C15) consist of three isoprene units, diterpenes (C20) consist of four isoprene units and the addition of more units will make up bigger terpene classes. The type of terpene produced also depends on the route of biosynthesis. The MEV pathway is more likely to produce sesqui- and triterpenes, whereas the MEP pathway produces mono- and diterpenes [34,37,43-45].

The topological network of the pathways in the model was constructed based on reaction stoichiometry and enzymatic mechanisms of known enzymatic reactions in the terpene biosynthetic pathway. Underlying biological information, including production and consumption rates, was used to generate the parameters of each HFPNe element that govern the behaviour of the model during simulations. Simulation and validation processes were then performed iteratively to produce an optimum system.

In this study, the possibility of discovering new alternatives for amplifying the production of terpenoids was the major goal. Therefore, a complete understanding of how the compounds are synthesised is vital.

## Results and Discussion

### Model overview

**Figure 2:**
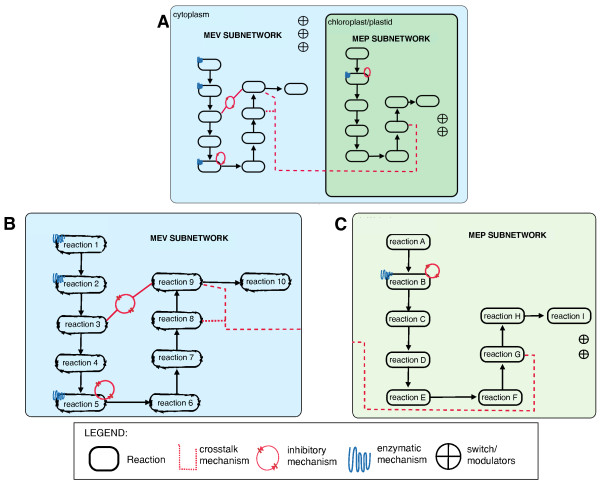
**Overall sketch of the terpenoid biosynthetic pathways**. The entire network is subdivided into MEV and MEP subnetworks, which are placed in different compartments. A rough sketch of the network is shown in A, with the MEV subnetwork on the left and the MEP subnetwork isolated in another compartment on the right. A clearer sketch of the MEV subnetwork is shown in B, and the MEP subnetwork is shown in C. The identified enzymatic and crosstalk mechanisms are shown in the sketches.

**Figure 3:**
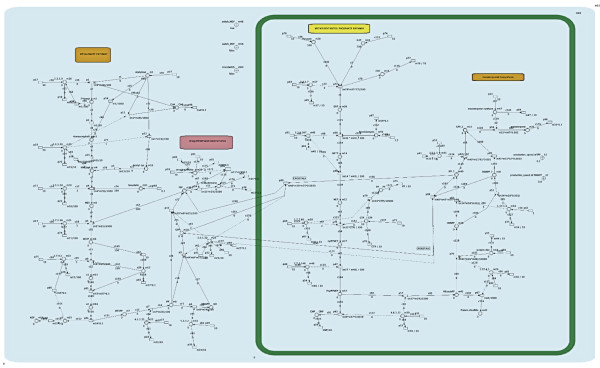
**The overall layout of the terpenoid biosynthetic pathway model**. All metabolites are represented using continous places whereas the regulatory components such as the on/off switches and parameter modulators are represented using generic places. Each transition entity represents a process such as phosphorylation, synthesis and degradation. Each substrate or intermediate is connected to the transition entity by a normal arc whereas an enzyme is connected to its transition entity using a test arc. The MEV subnetwork is on the left and the MEP subnetwork is secluded in a different compartment to depict that the pathway is operative in plastids of eubacterias or chloroplasts or plants.

This model consists of two subnetworks, the MEV and MEP subnetworks, which represent the respective terpene biosynthetic pathways. The study focuses on the synthesis of two classes of terpenes, sesquiterpenoids (the end product of the MEV pathway) and monoterpenoids (the end product of the MEP pathway). These two pathways are placed in different compartments in the model to reflect their different subcellular locations (Figure 2). All of the metabolites involved in the pathways are represented using HFPNe elements and are interconnected to each other based on their reaction stoichiometry and enzymatic mechanisms. The network consists of 101 continuous transitions representing various reactions and production/degradation processes; 66 places representing 61 metabolites; three on/off switches; and two parameter modulators. All metabolites were represented using continuous places, whereas generic places were used to create the regulatory components (on/off switches and parameter modulators). All metabolites were represented using continuous places whereas generic places were used to create the regulatory components (on/off switches and parameter modulators) (Figure 3, see also Additional file 1).

**Figure 4:**
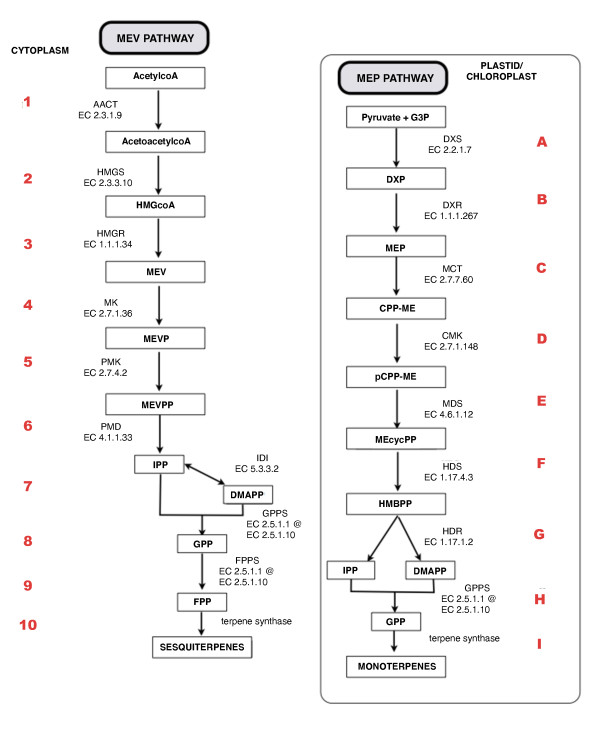
**The MEV and MEP pathway constructed using Cell Illustrator**. The pathways are numbered according to sequence, as shown in Tables 1 and 2.

The MEV subnetwork consists of ten main enzymatic reactions and two feedback loops, whereas the MEP subnetwork consists of nine main enzymatic reactions and a crosstalk mechanism that also involves the MEV subnetwork. The 19 main enzymatic reactions participating in both subnetworks are shown in Tables 1 and 2 and Figure 4. The behaviour of each reaction in the pathway is described using HFPNe element parameters such as speed of transition formulae, initial place values and threshold arc values [18,21,22,25]. To simulate the behaviour of the model in normal and/or perturbed conditions, the entities representing on/off switches are adjusted. The initial values of these entities can be manipulated to create different conditions during the simulation, leading to a better understanding of the pathway and pointing to reactions to be further manipulated. All simulations carried out in this study are based on known information retrieved from literature and database searches.

**Table 1:** Reactions involved in the MEV pathway (in order of occurrence)

Order^a^	Reaction	Enzyme	Main product^b^
1	acetylcoA + acetylcoA → acetoacetyl-coA + coA	acetylcoA C-acyltransferase (EC 2.3.1.9)	acetoacetyl-coA
2	acetoacetylcoA + acetylcoA + H_2_O → HMGcoA + coA	HMGcoA synthase (EC2.3.3.10)	HMGcoA
3	HMGcoA + 2NADPH + 2H^+ ^→ mevalonate + coA + 2 NADP^+^	HMGcoA reductase (EC 1.1.1.34)	MEV
4	MEV + ATP → MEVP + ADP	mevalonate kinase (EC 2.7.1.36)	MEVP
5	MEVP + ATP → MEVPP + ADP	phosphomevalonate kinase (EC 2.7.4.2)	MEVPP
6	MEVPP + ATP → IPP + PO_3 _+ CO_2 _+ ADP	MEVPP decarboxylase (EC 4.1.1.33)	IPP
7	IPP *eftrightarrow *DMAPP	IPP isomerase (EC 5.3.3.2)	DMAPP
8	IPP + DMAPP → GPP	GPP synthase (EC 2.5.1.1 atau EC 2.5.1.10)	GPP
9	IPP + GPP → FPP	FPP synthase (EC 2.5.1.1 atau EC 2.5.1.10)	FPP
10	FPP → sesquiterpenoid	sesquiterpene synthase	sesquiterpenes

^a ^Order of occurrence in the MEV pathway

^b ^Main product that serves as the substrate for the next reaction in the MEV pathway

**Table 2:** Reactions involved in the MEP pathway (in order of occurrence)

Order^a^	Reaction	Enzyme	Main product^b^
A	G3P + pyruvate → DXP + CO_2_	DXP synthase (EC 2.2.1.7)	DXP
B	DXP + NADPH + H^+ ^→ MEP + NADP^+^	deoxyxylulose phosphate reductoisomerase (DXR; EC1.1.1.267)	MEP
C	MEP + CTP → CPP-ME + diphosphate	MEP cytidyltransferase (EC 2.7.7.60)	CPP-ME
D	CPP-ME + ATP → pCPP-ME + ADP	CPP-ME kinase (EC 2.7.1.1480)	pCPP-ME
E	pCPP-ME → CMP + MEcycPP	MEcycPP synthase (EC4.6.1.12)	MEcycPP
F	MEcycPP + protein diol → HMBPP + H_2_O + protein disulfide	HMBPP synthase (EC 1.17.4.3)	HMBPP
G	HMBPP + NADPH + 2H^+ ^→ IPP + DMAPP + H_2_O + NADP^+^	HMBPP reductase (EC 1.17.1.2)	IPP dan DMAPP
H	IPP + DMAPP → GPP	GPP synthase (EC 2.5.1.10 atau EC 2.5.1.1)	GPP
I	GPP → monoterpenoid	monoterpene synthase	monoterpenes

^a ^Order of occurrence in the MEP pathway

^b ^Main product that serves as the substrate for the next reaction in the MEP pathway

Based on the introduction of inhibitors and increments of metabolite concentration in both pathways, together with the regulation of the three switch entities, we here summarise five simulation conditions:

1) normal production of sesquiterpenes through the MEV subnetwork, with the MEP subnetwork and all inhibitory processes deactivated;

2) normal production of monoterpenes through the MEP subnetwork, with the MEV subnetwork and all inhibitory processes deactivated;

3) overexpression of ATP in the fifth reaction of the MEV subnetwork, with the MEP subnetwork and other inhibitory processes deactivated;

4) overproduction of FPP in the ninth reaction of the MEV subnetwork, with the MEP subnetwork and other inhibitory processes deactivated;

5) establishment of a crosstalk mechanism with the MEP subnetwork activated but with the MEV subnetwork as well as other inhibitory processes deactivated.

### Simulation and Validation

The simulation and validation processes, as well as the developmental stages of the model performed in this study, were carried out using Cell Illustrator https://cionline.hgc.jp. The model was validated against known data and information obtained from extensive literature and database searches. Simulation results are returned as concentrations (unit) versus Petri net time (pt) graphs. Here, we discuss three of the five simulated conditions carried out in the study.

#### a. Overproduction of ATP

**Figure 5:**
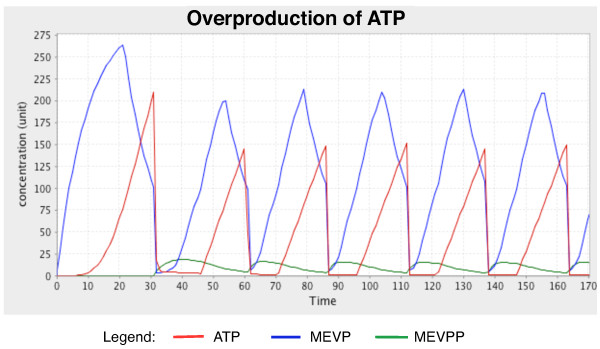
**Concentration (unit) changes in metabolites involved in the feedback loop mechanism over time (pt)**. Oscillations between the concentrations describe how MEVPP production halts when high concentrations of ATP are present and resumes when the concentration of MEVP increases.

In the fifth reaction of the MEV pathway, phosphomevalonate kinase (PMK; EC 2.7.4.2) catalyses the production of mevalonate diphosphate (MEVPP) and ADP from mevalonate-5-phosphate (MEVP) and ATP (Table 1) [46-48]. This reaction acts in a bi bi (also known as two-substrate/two-product) sequential enzymatic mechanism with respect to the two substrates; MEVP first binds to the free enzyme, followed by ATP; the enzyme then releases the main product and finally ADP [46]. Studies have shown that high concentration of ATP halts the production of MEVPP and thus blocks the entire pathway from continuing to synthesise other intermediates. ATP acts as a competitive inhibitor, binding to both sites of the free enzyme and forming a dead-end complex. This obstructs the binding of MEVP to the enzyme and impedes the downstream reactions in the pathway. However, it was also shown that by increasing the concentration of MEVP, the inhibitory effects of ATP could be reversed. Higher concentrations of MEVP inhibit the synthesis of ATP, lowering the concentration of ATP to a manageable level. This allows the synthesis of MEVPP, as well as downstream reactions, to resume (Figure 5) [46].

To simulate this mutually antagonist inhibitory feedback loop, the 'MEV_switch' component is set to 'true' and the other two switches are set to 'false.' The parameter that controls the production of ATP is adjusted to a higher value of 30 unit/pt from 10 unit/pt, resulting in overproduction of ATP by 3-fold and activates the inhibitory arc. The threshold value for the inhibitory arc that suppresses the production of ATP is set to 80 units to allow the inhibitory effect to take place.

#### b. Overproduction of FPP

**Figure 6:**
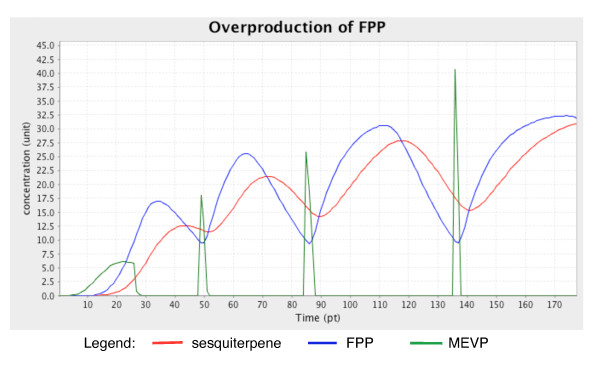
**Concentration (unit) changes in metabolites involved in the feedback loop mechanism caused by overproduction of FPP over time (pt)**. The simulation successfully describes how high concentrations of FPP affect the production of MEVP. The oscillatory behaviour also affects the production of sesquiterpenes.

The second feedback loop identified in the MEV pathway is a positive feedback mechanism. It is initiated when FPP levels become high enough to inhibit the catalytic activity of mevalonate kinase (MK; EC2.7.1.36). MK catalyses the fourth reaction in the MEV pathway, phosphorylation of mevalonate to produce mevalonate-5-phosphate (MEVP) (Table 1). The inhibitory activity of FPP halts the phosphorylation of mevalonate, thus slowing the downstream processes, including the production of FPP itself [49,50]. This will return FPP concentration to its normal level, releasing its inhibitory effect on MK (Figure 6).

To simulate this behaviour, the 'MEV_switch' component is set to 'true' and the two other switches are set to 'false.' No other inhibitory processes except the inhibitory arc connecting FPP to the catalytic activity of MK are activated. The threshold value of the arc is set to 10 units from a higher value of 100 units to allow the inhibitory effect to take place. The simulation result shows an oscillatory behaviour of the concentrations of sesquiterpene, FPP and MEVPP over time (Figure 6).

#### c. Crosstalk

**Figure 7:**
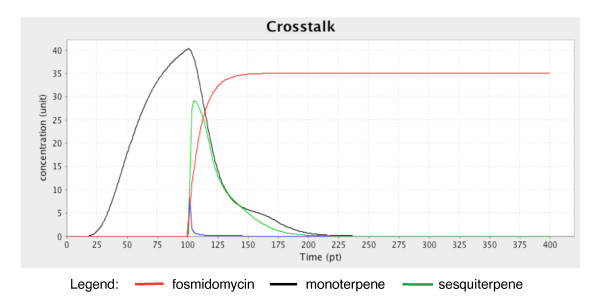
**Concentration (unit) changes in metabolites affected by the crosstalk mechanism over time (pt)**. When the concentration of fosmidomycin increases over time, monoterpene production decelerates at 100 pt; however, a slight increase in sesquiterpene concentration is observed.

**Figure 8:**
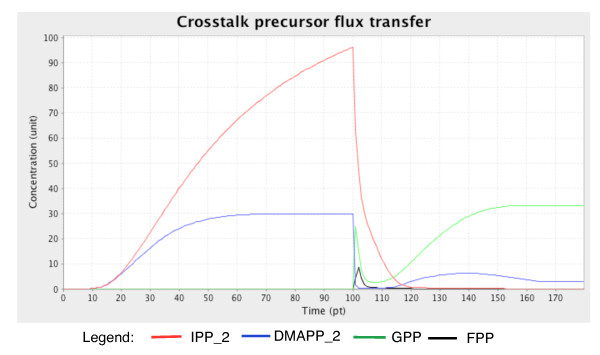
**Concentration (unit) changes in levels of the main precursors involved in the crosstalk mechanism between both pathways over time (pt)**. IPP_2 and DMAPP_2 are precursors from the MEP subnetworks, whereas GPP and FPP serve as precursors in the MEV subnetwork. The transfer of fluxes from IPP_2 and DMAPP_2 to the MEV subnetwork enables GPP and FPP to be synthesised and thus produce sesquiterpenes.

A crosstalk mechanism between the MEP and MEV subnetworks is established with the introduction of the inhibitor fosmidomycin. This inhibitor suppresses the catalytic activity of deoxyxylulose-5-phosphate reductoisomerase (DXR; EC1.1.1.267). Suppression of DXR activity reduces the rates of the downstream reactions in the MEP pathway, affecting the production of monoterpenes (Figure 7). However, the inhibition also triggers a flux of precursors (IPP and DMAPP) from the MEP pathway to the MEV pathway, resulting in increased sesquiterpene concentration despite the inactivation of the MEV pathway (Figure 8) [51]. This event is described in the model by assigning function values to the transition elements of the components that are involved in this mechanism. These functions are then regulated by a switch component called 'crossSwitch.'

To simulate the crosstalk mechanism, the 'MEV_switch' component is set to 'false.' The 'MEP_switch' component is set to 'true' while the 'crossSwitch' component is given a script value. The script value enables the activation of the 'fosmidomycin' component after 150 pt, thereby establishing the crosstalk mechanism. The simulation result successfully described oscillatory changes in the concentrations of the metabolites involved over time.

## Conclusion

The simulation and validation processes performed using the model are consistent with known biological information and data. The intuitive approach introduced by the HFPNe technique enables intricate modelling tasks to be viewed and solved in a graphical perspective. The model serves as a tool for better understanding the reactions involved in both pathways and how they affect each other. Perturbations performed on the model provide insight into the effects of manipulation of a single reaction on the whole network and thus should facilitate both industrial and biomedical terpenoid bioengineering.

## Methods

### Information and data acquisition

The biological information and data needed to construct this model included information on the substrates, products and enzymes involved in the MEV and MEP pathways, as well as detailed enzymatic reaction information such as reaction stoichiometry and enzyme mechanisms. Consumption and production rates were also taken into consideration in developing the dynamic model. This information was used to design the model layout and parameter assignments for each of the HFPNe elements. The data were obtained through the use of extensive literature and biological databases including KEGG, BRENDA, ENZYME, IUBMB, MetaCyc and PATHWAY DATABASE [23,52-56].

### Model template design and parameter assignment

The HFPN elements (place, transition and arc) represent the metabolites and processes that comprise the MEV and MEP biosynthetic pathways. Continuous places represent the substrates, products, inhibitors and enzymes in both subnetworks, whereas continuous transitions represent biological processes such as catalysis, phosphorylation and degradation. Generic places represent the on/off switches and parameter modulators. The normal arcs then connect places (enzyme, substrate, product) to transitions while inhibitory arcs connect inhibitors to their prospective inhibited processes. All these components were arranged according to the reaction stoichiometry and enzymatic mechanism in their order of occurrence in the respective pathways (Tables 1 and 2).

Each of the HFPNe elements possesses different attributes that characterise its function in the model. Parameter values are assigned to each of these attributes to control the behaviour of the model during simulation. These values are generated based on the biological information obtained and are translated into virtual concentration values (initial values of places), ordinary differential equations (speed formulas of transitions), numeric values (threshold of normal and inhibitory arcs) and scripts (on/off switches) [10,18,22-25].

### Iterative model development

The test-driven models were simulated and validated against known data obtained through literature searches. Simulations were carried out under five different conditions as explained in the Results and Discussion section. Simulation results were displayed as concentration (unit) versus time (pt) graphs. Petri net time (pt) indicates virtual Petri net time units that do not correspond to real time; concentration also is given in general concentration units (unit) that do not specifically correspond to actual concentration units such as mM and μM. The changes in metabolite concentrations (unit) over time predicted by each simulation were validated against known biological data to identify gaps and inconsistencies. Uncorrelated biological behaviours were re-examined and previous developmental steps (topological design and parameter assignment) were repeated. The simulation process was then re-executed and revalidated against known biological information. The entire process was carried out iteratively in order to rule out inconsistencies and obtain an optimum system.

## List of abbreviations

ADP: (adenosine diphosphate); ATP: (adenosine triphosphate); DMAPP: (dimethylallyl diphosphate); DXR: (deoxyxylulose-5-phosphate reductoisomerase); EC: (enzyme commission); GPP: (geranyl diphosphate); HDN: (Hybrid Dynamic Petri net); HFPNe: (Hybrid Functional Petri net with extension); HPN: (Hybrid Petri net); IPP: (isopentenyl diphosphate); FPN: (Functional Petri net); FPP: (farnesyl diphosphate); pt: (Petri net time); MEP: (methylerythritol-5-phosphate); MEV: (mevalonate); MEVP: (mevalonate-5-phosphate); MEVPP: (mevalonate diphosphate); MK: (mevalonate kinase); ODE: (ordinary differential equation); PMK: (phosphomevalonate kinase).

## Authors' contributions

AHH performed the research and drafted the manuscript. ZAMH formulated the study, gave valuable insights, participated in its design and helped to draft and refine the manuscript. All authors read and approved the final manuscript.

## Supplementary Material

Additional file 1 **Two simulation files to complement Figure 3**. Click here for file
